# An artificial intelligence based abdominal aortic aneurysm prognosis classifier to predict patient outcomes

**DOI:** 10.1038/s41598-024-53459-5

**Published:** 2024-02-09

**Authors:** Timothy K. Chung, Pete H. Gueldner, Okechukwu U. Aloziem, Nathan L. Liang, David A. Vorp

**Affiliations:** 1 Department of Bioengineering, University of Pittsburgh, Pittsburgh, PA USA; 2 School of Medicine, University of Pittsburgh Medical Center, Pittsburgh, PA USA; 3 Division of Vascular Surgery, Department of Surgery, University of Pittsburgh Medical Center, Pittsburgh, PA USA; 4 Department of Surgery, University of Pittsburgh, Pittsburgh, PA USA; 5 McGowan Institute for Regenerative Medicine, University of Pittsburgh, Pittsburgh, PA USA; 6 Department of Chemical and Petroleum Engineering, University of Pittsburgh, Pittsburgh, PA USA; 7 Department of Cardiothoracic Surgery, University of Pittsburgh, Pittsburgh, PA USA; 8 Clinical & Translational Sciences Institute, University of Pittsburgh, Pittsburgh, PA USA; 9 Department of Mechanical Engineering and Materials Science, University of Pittsburgh, Pittsburgh, PA USA; 10 Center for Vascular Remodeling and Regeneration, University of Pittsburgh, Pittsburgh, PA USA; 11 Bioengineering, Cardiothoracic Surgery, Surgery, Chemical and Petroleum Engineering and the Clinical and Translational Sciences Institute, Center for Bioengineering, University of Pittsburgh, 300 Technology Drive, Suite 300, Pittsburgh, PA 15219 USA

**Keywords:** Biomedical engineering, Computational models, Image processing, Machine learning, Predictive medicine

## Abstract

Abdominal aortic aneurysms (AAA) have been rigorously investigated to understand when their clinically-estimated risk of rupture—an event that is the 13th leading cause of death in the US—exceeds the risk associated with repair. Yet the current clinical guideline remains a one-size-fits-all “maximum diameter criterion” whereby AAA exceeding a threshold diameter is thought to make the risk of rupture high enough to warrant intervention. However, between 7 and 23.4% of smaller-sized AAA have been reported to rupture with diameters below the threshold. In this study, we train and assess machine learning models using clinical, biomechanical, and morphological indices from 381 patients to develop an aneurysm prognosis classifier to predict one of three outcomes for a given AAA patient: their AAA will remain stable, their AAA will require repair based as currently indicated from the maximum diameter criterion, or their AAA will rupture. This study represents the largest cohort of AAA patients that utilizes the first available medical image and clinical data to classify patient outcomes. The APC model therefore represents a potential clinical tool to striate specific patient outcomes using machine learning models and patient-specific image-based (biomechanical and morphological) and clinical data as input. Such a tool could greatly assist clinicians in their management decisions for patients with AAA.

## Introduction

Abdominal aortic aneurysm (AAA) is an irreversible localized dilatation of the aorta that, if left untreated, may lead to rupture, an event that is the 13th leading cause of death in the United States^1^. Upon diagnosis of AAA, a clinician will intervene based on a single one-dimensional measurement—the maximum diameter criteria (5.0 cm for women, 5.5 cm for men)—when the risk of rupture is thought to be higher than the risk of repair. However, a significant number of small (sub-threshold) AAA have been reported to rupture (between 7 and 23.4%)^1–3^, suggesting that the maximum diameter criteria have high false-negative rates. Additionally, there is an unknown false positive rate for patients that would die from other causes before their aneurysm would rupture, regardless of aneurysm size. These considerations highlight the deficiencies of the more than 60-year-old one-size-fits-all maximum diameter criterion to reliably assess AAA patient prognosis.

Assessing the biomechanical status of AAA—with a growing number of studies over the past ~ 25 years—has been motivated by viewing aneurysm rupture as a biomechanical event wherein the transmural wall stresses acting on the AAA exceeds the strength of its wall^1,4^. These studies have included experimentally assessing the material properties of degenerated AAA wall tissue^5,6^ and commonly involved intraluminal thrombus^7–9^ as well as comprehensive patient-specific computational finite element models to assess wall stress distributions^7,10–14^. In addition, a rupture potential index was introduced by our group as a local ratio of calculated wall stress and estimates of wall strength^7,15^. Major limitations of all biomechanical-based studies include high variations in the stress analysis approaches of AAA models and the fact that nearly all of the aneurysms that have been analyzed in the literature were “clinically-sized” (greater than in the maximum diameter criteria^16^), narrowing relevance to only post-threshold, end-stage AAA. In addition to the lack of significant clinical translation of biomechanical tools in clinical management of AAA to date, there has not been a single conclusive study reporting a causal effect between elevated wall stress and rupture site, one group attempted to show this effect, but was unable to find a relationship^17^. Further interrogation of retrospective imaging datasets and clinical data is necessary to draw out potential biomarkers that can classify and striate patient outcomes on whether an aneurysm is at risk of rupture or in need of eventual clinical intervention.

Quantification of AAA morphometrics   has also been utilized to capture and propose one-, two-, and three-dimensional indices^11,18^ to investigate the potential correlation between shape and AAA status^19–21^. These have included various diameter ratios^22^, AAA wall surface area^19^, volume^21^, asymmetry^18^, and tortuosity^19^, intraluminal thrombus (ILT) thickness^9^ and volume, and measures of local surface curvatures (Gaussian and mean principal)^21^. However, these studies have again been limited in that they have relied on clinically sized aneurysms due to the availability of medical imaging data.

The advent of artificial intelligence (AI) tools, mainly machine learning (ML) algorithms, provide the possibility of diagnosis and guidance on clinical management for various diseases^23,24^. Clinical decision support and management is critical in AAA prognosis as it has been reported that the average 4-year cost of surveillance is $40,528 and that smaller-sized aneurysms are still at risk of rupture^25^. Therefore, such AI/ML-based tools for AAA prognostics in particular could have a very big impact. Studies using clinical inputs paired with ML have shown promise in providing an objective clinical decision support tool^23,26^, but there is a need to incorporate image-derived metrics such as the biomechanical status and morphology to improve models and clinical relevance.

In this study, a novel aneurysm prognosis classifier (APC) was constructed based on a ML model that was to striate patient outcomes for stable, repair, and ruptured AAA. A rigorous imaging-based analysis was performed to compute morphological and biomechanical indices to use alongside key clinical indices for use in the ML classification models. The models were then trained using all three sets of indices along with known patient outcomes (stable, repair, and rupture). Additional analysis is presented that provides insight to the importance of pairing imaging-based studies (biomechanical and morphological) with clinical indices towards the development of a clinical decision support tool for patient prognosis of AAA.

## Methods

### Clinical and image data

This study was approved by the ethics committee of the University of Pittsburgh. The study methods, protocols, and data access were performed in accordance with the University of Pittsburgh Institutional Review Board (IRB) that are specific to the guidelines and regulations provided by the Human Research Protection Office within the Department of Health and Human Services under #STUDY19060084 to analyze the retrospective database. Informed consent was given by each and/or their legal guardian(s) by the University of Pittsburgh Medical Center. The retrospective dataset generated and/or analyzed during the current study are not publicly available due to limitations of the scope of the IRB but are available from the corresponding author on reasonable request.

**Figure 1:**
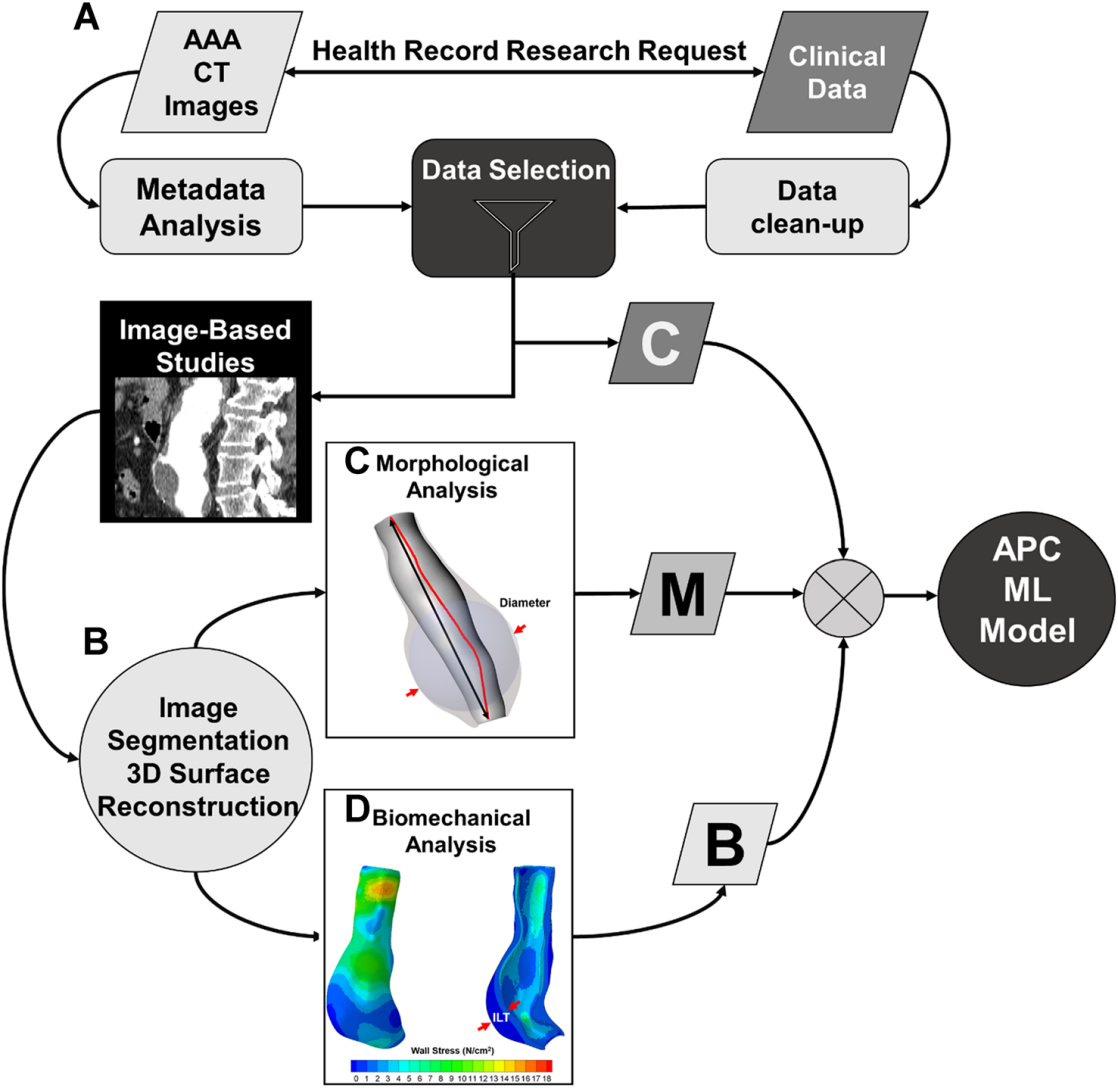
Pipeline to prepare the tabulated APC input datasets. (**A**) De-identified clinical and imaging data acquired from R3 is followed by image segmentation (**B**), 3D reconstruction and morphological analyses (**C**) and biomechanical analysis (**D**). The output from this pipeline includes a cleaned-up clinical dataset *C*, morphological metrics *M*, and biomechanics-based metrics *B* that are used as inputs to the APC machine learning model.

An anonymized set of clinical and imaging data was delivered via Globus cloud services (Argonne National Laboratory, Chicago, IL) by the Health Record Research Request (R3) in collaboration with the University of Pittsburgh Medical Center and the Department of Biomedical Informatics at the University of Pittsburgh (Fig. 1A). Longitudinal data from 381 unique AAA patients from a minimum of two different time points and with known clinical outcomes were provided by R3. The two unique time points was used as a filtering criterion to ensure that a clinical outcome was known (repair or rupture) and stable if the last available medical image did not have clinical intervention or a rupture event (i.e., a single timepoint would not inform the study team of patient outcomes). There was a total of 352 stable aneurysms (defined as not having intervention or rupture during their longitudinal study), 16 clinical interventions, and 13 rupture events in the cohort. The first time point used for each patient was chosen based on a filtering criterion prioritizing the highest quality CT image set available. The clinical data (see Supplementary Table 1) included patient demographics, pharmaceutical use, and co-morbidities. The binary clinical indices were binary encoded and used ‘1’ for when a condition was present and ‘0’ for the absence of the condition for all co-morbidities. The clinical dataset was pre-processed using a custom python script to extract and organize clinical variables into columns for each patient. The known clinical outcomes were encoded to denote ‘0’ for stable, ‘1’ for repaired, and ‘2’ for ruptured aneurysms.

### Image segmentation pipeline with automated U-NET and semi-automated image processing

A U-NET image classifier was recently reported by our group to automatically segment AAA regions of interest from medical images^27^. In that work, training was performed using a custom python script for U-NETs to run on both a local workstation optimized for multi-GPU training with four NVIDIA (NVIDIA Inc., Santa Clara, CA) A5000 ’s graphics cards and an Amazon Web Services Elastic Computing Node EC2 (Amazon Inc., Seattle, WA). The trained and validated image classifier was implemented in the current workflow to identify the AAA wall, ILT, and lumen (Fig. 1B)^27^. Semi-automated image segmentation using a custom in-house MATLAB script was performed for any image sets that had poor aneurysm wall and/or lumen boundary isolation using the U-NET.

### 3D surface reconstruction and morphological analyses

The segmented wall, lumen, and ILT regions from axial slices were converted into a binary mask for further processing to create 3D surface reconstructions and eventual meshing for computational analysis described further in the next section. Point clouds for each axial slice were created from the outer boundary for the wall and inner boundary for the lumen. The spacing between axial slices was calculated from the respective positions within the image stack and the regions were scaled from the pixel to millimeter (mm) conversion ratio found in the DICOM header file. Point clouds for the wall and lumen were meshed using Poisson surface reconstruction by computing the normal of each vertex with a neighborhood of 25 vertices^28^. Morphological analysis to yield one-, two-, three-, and higher dimensional indices for each aneurysm in the dataset was performed on the 3D reconstructed surfaces (Fig. 1C). The morphological indices that were measured were chosen based on previously reported literature and are found in Supplementary Table 2.

### Finite element analysis and extraction of biomechanical indices

The finite element analysis (FEA) used in this study followed well-established methodology and incorporates previously published, experimentally measured material properties of the aneurysm wall and ILT^6,7,9^. The initial surface from the point clouds were converted into polysurfaces (for the lumen and wall) that were imported into ANSYS ICEM (ANSYS Inc., Cannonsburg, PA) where the geometries were meshed with both 2D shell elements (for the wall) and 3D volumetric elements (for the ILT). A uniform AAA wall thickness of 1.9 mm was assumed^7,27^. The computational mesh was constructed using 2D shell elements (S3R) for the wall and 3D tetrahedral elements (C3D4) for the ILT. The AAA wall was assumed to be hyperelastic and anisotropic^7^, while the ILT was assumed to be hyperelastic and isotropic^9^. An established, validated hyperelastic isotropic material model was used for the ILT^29^, and an established anisotropic model was used for the AAA wall material^7^ using a user-defined function. The isotropic ILT model uses a material model consisting of two parameters c_1_ = 2.6 N/cm^2^, and c_2_ = 2.6 N/cm^2^, respectively^7,9,29^. The biaxial behavior was previously modeled and descried by Vande Geest et al.^7^ where the strain energy assumption is defined by the following form (1):

1 $$W = b_{o} \left( {e^{{\frac{1}{2}b_{1} E_{\theta \theta }^{2} }} + e^{{\frac{1}{2}b_{2} E_{LL}^{2} }} + e^{{b_{3} E_{\theta \theta } E_{LL} }} + e^{{\frac{1}{2}b_{4} E_{\theta L}^{2} }} + e^{{b_{5} E_{\theta \theta } E_{\theta L} }} + e^{{b_{6} E_{LL} E_{\theta L} }} - 6} \right)$$

Where b_0_, b_1_, b_2_, and b_3_ are 0.14, 477.0, 416.4, and 408.3 kPa, respectively^6^. The strain energy terms involving the constants b_4_, characterize material shear and b_5_, and b_6_ the shear-normal behaviors (included for completeness, but is not required for fitting experimental stress–strain data)^7,30^. The distal and proximal ends of the AAA were constrained in the x, y, and z directions and an ideal systolic pressure of 120 mmHg was applied to the surface of the lumenal elements. All simulations were performed in Abaqus Standard (implicit mode) with Microsoft Visual Studio 2017 (Microsoft Inc., Redmond, WA) and Intel Fortran Compiler (Intel Inc., Santa Clara, CA) employing a user-defined function to prescribe the anisotropic material properties of the AAA wall^7^ (Fig. 1D). Lastly, von Mises wall stresses were computed (nodal average) and reported for both peak and mean wall stresses.

### Statistical analysis of tabulated variables

The Kaplan–Meier estimator was used to model the survivability in our patient cohort to measure the number of patients that underwent clinical intervention or had rupture events. Correlation matrices between individual input variables and patient outcomes were constructed using a MATLAB code to qualitatively visualize and quantitatively assess whether positive or negative correlation relationships existed. Additionally, an ANOVA statistical test was performed for each variable to assess differences between clinical outcome groups using the three possible comparisons; i.e., stable vs. repair, stable vs. rupture, and repair vs. rupture. The primary purpose of this analysis was to determine whether a non-AI-based approach could discriminate patient outcomes for each group as well as the APC ML model.

### Machine learning model training and testing

**Figure 2:**
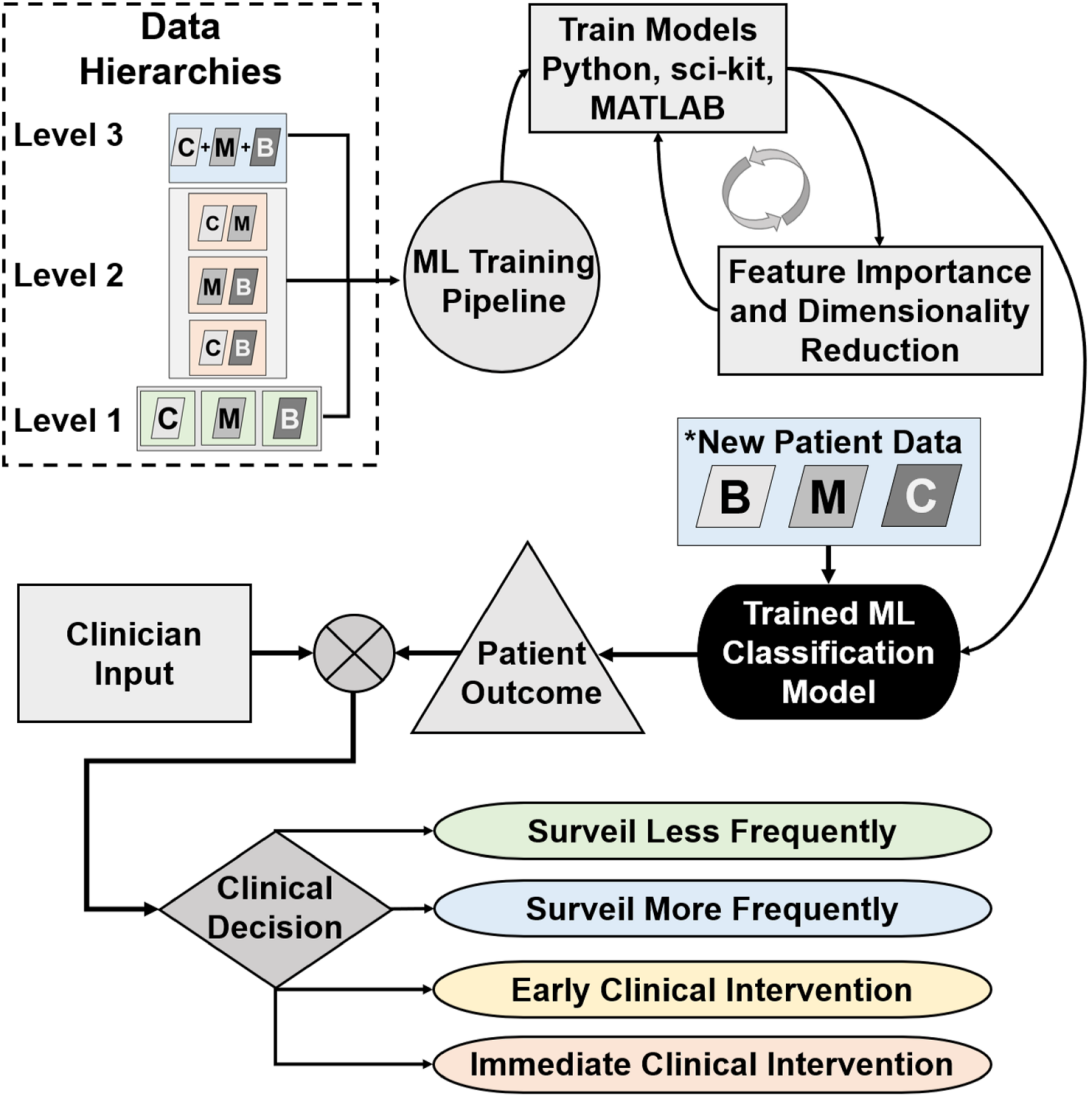
There were different combinations of ML training (Levels 1–3) that were used for three categories: clinical (C), biomechanical (B), and morphological (M). The ML models were iteratively trained using various algorithms to reduce the number of variables for the finalized classification model. For the holdout validation, a testing dataset not seen by the ML training set was used to validate and classify patient outcomes. The proposed use for APC is to weigh in clinician input to come to a finalized clinical decision that could reduce the frequency of surveillance, offer early clinical intervention, or recommend immediate clinical intervention.

A dataset was prepared for APC ML model training by organizing the three categories of indices (biomechanical, morphological, and clinical) into a comma delimited file with the last column corresponding to each patients’ clinical outcome. Training was performed using ensemble boosted tree algorithms in MATLAB and python libraries using sci-kit/sklearn^31^ and XGBoost^32^. Training was performed using combinations of categories to elucidate the importance of features (Fig. 2). The lowest level of training (Level 1) was performed separating each category (biomechanical, morphological, and clinical) individually with patient outcomes. Intermediate level of training (Level 2) was performed using paired categories: clinical and biomechanical, clinical and morphological, and biomechanical and morphological with clinical outcomes. The highest level of training (Level 3) combined all categories with each patient’s respective clinical outcome. Training was performed using internal seven-fold cross-validation techniques reserving 20% of the data for testing. Receiver operator characteristic (ROC) curves were generated for each clinical outcome and the area under the curve was calculated that represents the discriminability of the classification model. Confusion matrices and ROC curves were generated for each training level and the area under the curve (AUC) was calculated to inform the ability of each model to predict outcomes (stable, repair or ruptured). Feature importance was computed for all trained models with their respective weights to truncate the number of variables used for training and testing. A threshold of 0.02 for feature importance was used to reduce the number of overall variables used in the classification model (i.e., weights below 0.02 were not considered in the final model).

To reduce the model bias or potential overfitting effects, a hold-out approach was performed by having a separate training and testing dataset. The rationale for this is that the algorithm that is produced should not have ‘seen’ any of the testing data during the training phase. Although the purpose of internal cross validation through simulating various splits, the hold-out validation model allows for a ‘real-world’ look at inputting new data into the trained model. To this end, the hold-out validation approach reserved ~ 80% of the entire dataset (n = 285 for the stable group, n = 11 for the repair group, n = 9 for the ruptured group) exclusively for training and ~ 20% (n = 68, n = 4, and n = 4, respectively) reserved for testing using a randomization procedure within each group. The hold-out validation method was applied to the combined biomechanical, morphological, and clinical categorical indices, rather than training for Levels 1–3. The testing dataset was input into the trained models to assess the ability to predict patient outcomes. Lastly, we compared the classification results of stable, repair, and rupture groups against the maximum diameter criterion for female and male subjects to further interrogate the model against the current clinical standard.

## Results

**Figure 3:**
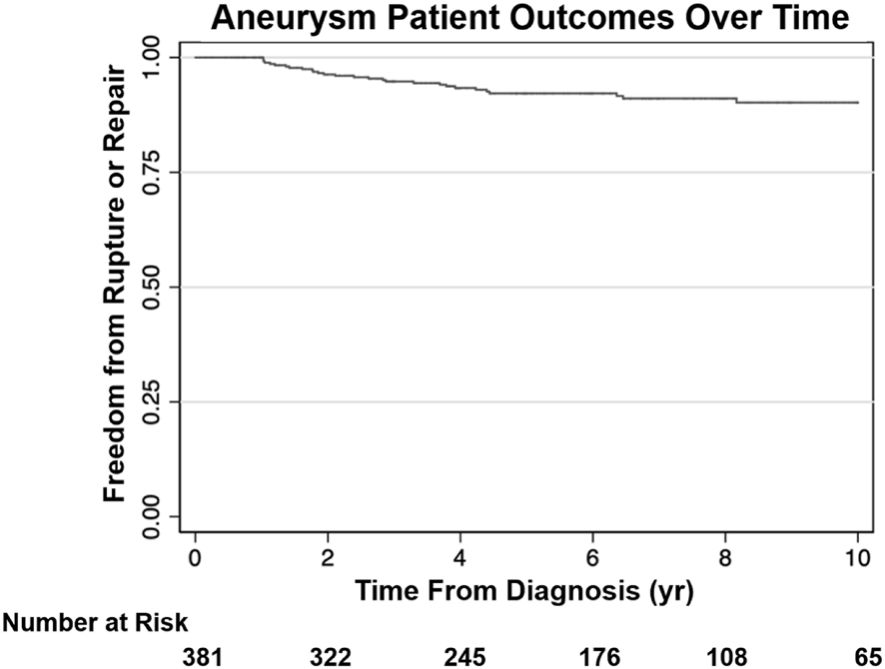
Survivability analysis of the AAA patient cohort displays that 83% of patients survived 10 years after being diagnosed with AAA. Number of aneurysms that were considered in each time point indicated under number at risk.

The survival curve shows that no patients in our study had surgical repair or rupture within the first year of aneurysm discovery, where the number of patients at risk was 17% or 65 patients over a 10-year period (Fig. 3). Additionally, the survival curve reveals that 4 years after aneurysm discovery, the rate of repair or rupture stabilizes (i.e., levels-off without further events), and 83% of the studied patients made it 10 years without either rupture or need for repair. The average times-to-event for repair and rupture were 2.50 ± 1.55 years and 3.25 ± 2.15 years, respectively. It was also found that stable cases went without treatment for an average duration of 6.19 ± 3.47 years for that cohort.

Supplementary Table 3 reports the general demographics and clinical indices for each group of patients (stable, repair, and rupture). The full patient cohort was on average 67.4 ± 9.18 years old and expressed a variety of common AAA comorbidities. Supplementary Table 4 provides statistical comparisons of biomechanical and morphological data between each group pairs (stable vs. repair, stable vs. rupture, repair vs. rupture). A correlation matrix was created for the full list of variables (Supplementary Fig. 1) and the truncated list of variables based on the Gini importance threshold of 0.02 used during the training phase for the classification model (Supplementary Fig. 2). There were no variables that independently correlated (positively or negatively) with patient outcome.

AUC values for predictions of stable, repair, and rupture of the various models for Levels 1, 2 and 3 are shown in Table 1. For example, for Level 1 training, the AUC for the “Repair” group was 0.65 for clinical data training alone, 0.81 for biomechanical data training alone, and 0.73 for morphological data training alone. For the Level 2 training, the AUC for the “Repair” group was 0.82 for both clinical and biomechanical data training and clinical and morphological data training, and 0.76 for biomechanical and morphological data training. However, the classification models that underwent Level 2 training failed to predict rupture events demonstrated in Supplementary Fig. 3, where no predictions were seen in the confusion matrices. The ROC curves that were generated represent the ability of the model to discern each outcome, and a higher corresponding AUC (closer to 1) provides how well the model is performing for a given patient outcome. For the Level 3 trained ML model, the AUC was 0.88 when predicting stable aneurysms, 0.87 when predicting clinical intervention, and 0.79 when predicting rupture (Supplementary Fig. 3). The confusion matrices at each level of training revealed an inability to predict repair and rupture events for Levels 1 and 2 training (Supplementary Fig. 3). Level 3 training was able to accurately predict patient aneurysm events (2.6% for repair and 1.3% rupture, even with the low number of patients with these outcomes), but had a higher level of misclassification (e.g., predicting 16.1% as rupture when it was stable). It was also shown that 0.78% of the patients were classified as ‘stable’ when they ruptured (Supplementary Fig. 3). Feature importance was calculated using the Chi-squared method and ANOVA reporting the top 15 variables (Supplementary Fig. 4).

**Table 1:** Training and testing AUC results of the final model with truncated variables.

		Training			Testing		
		Stable	Repair	Rupture	Stable	Repair	Rupture
Level 1	C	0.56	0.65	0.50	0.74	0.66	0.80
	B	0.77	0.81	0.67	0.79	0.80	0.71
	M	0.73	0.73	0.71	0.84	0.82	0.81
Level 2	C + B	0.79	0.82	0.74	0.94	0.92	0.89
	C + M	0.81	0.82	0.76	0.90	0.88	0.86
	B + M	0.81	0.76	0.95	0.90	0.81	0.86
Level 3	C + B + M	0.88	0.87	0.79	0.94	0.92	0.93
Split Data	C + B + M	0.82	0.78	0.83	0.90	0.80	0.91

Levels 1–3 use all patients for training and testing. The “split data” row used a subset of data reserved for training and testing.

**Figure 4:**
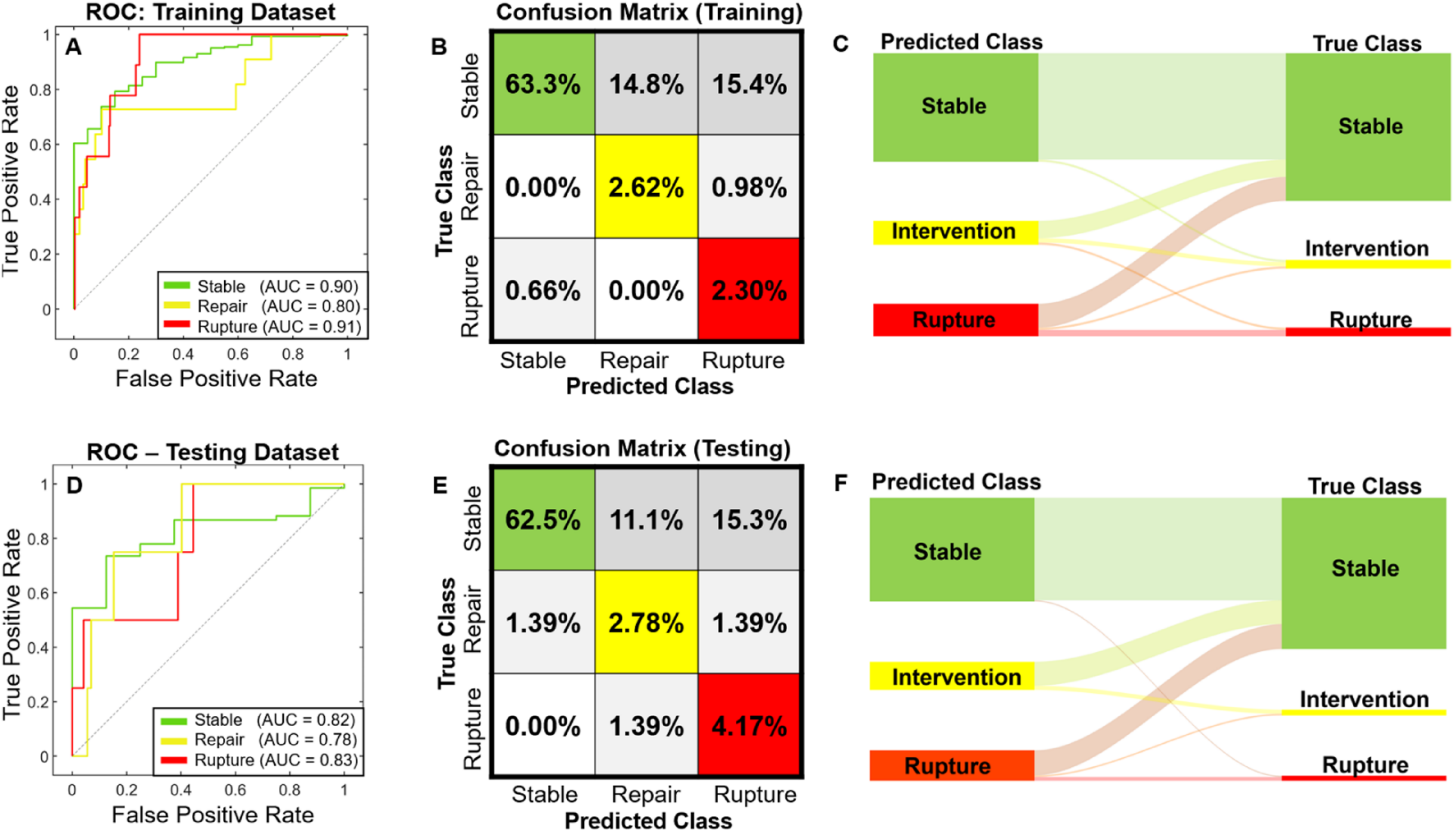
(**A**) ROC for training (n = 305), (**B**) internal cross validation of the training models, (**C**) Sankey diagram of the training dataset (predictions and true class), (**D**) ROC for holdout testing (n = 76), (**E**) holdout testing of the trained model, (**F**) Sankey diagram of testing dataset (predictions and true class).

For the hold-out validation approach (splitting the training and testing dataset prior to training), the AUC values for the stable, repair, and rupture groups were 0.82, 0.78, 0.83, respectively, for the training dataset, and 0.90, 0.80, and 0.91, respectively, for the test set (Fig. 4). The hold-out testing dataset included a combination of stable, repair, and rupture patients (n = 68, n = 4, and n = 4, respectively). The APC model classified 50% of the rupture accurately but misclassified a single patient as stable and another as repair, where the rupture subset was all male and a diameter of 5.29 ± 0.25 cm. The APC model classified 75% of the repair accurately but misclassified a single patient as rupture, where the repair subset was all male and had a diameter of 5.09 ± 0.32 cm. Lastly, the APC model classified 72.1% (n = 49) of the stable cases accurately but misclassified 10.3% (n = 7) as repair and 17.6% (n = 12), where 92.6% were male and 7.4% female with an average diameter of 4.56 ± 1.4 cm.

## Discussion

A trained ML classification model was developed here using image-based indices (biomechanical and morphological) and clinical indices with the ability to predict patient outcomes that included stable, intervention, and rupture patient outcomes. It is important to note that the ML classifier was able to pick up small changes within the cohort to correctly predict repair and rupture events. Three levels of training were performed using each type of indices independently (Level 1), paired combinations of types (Level 2), and all three types of indices together to classify and striate patient outcomes (Level 3). It was found that Level 1 training was generally poor without the proper discriminability to predict all classes of patient outcomes. Using clinical data alone resulted in the lowest performing classification model highlighting the limitations of this data category. Conversely, the classification algorithms were seen to greatly improve when using image-base indices derived from biomechanics and morphological quantification. Given that the Level I and II training were poor using the internal cross-validation approach, the holdout validation was not performed.

Biomechanical and morphological analyses of AAA have been previously studied to identify potential biomarkers for rupture prediction^1,13,15,33–35^. Peak and mean wall stresses have been shown in some of these studies to be elevated in symptomatic and ruptured aneurysms^12–14,30^. Further efforts to combine ML methods with biomechanical and morphological techniques have been utilized to predict rupture events^36,37^. The previous image-based studies suffer from a general bias of using larger, clinical-sized aneurysms that are near or exceed the maximum diameter criterion reducing the potency of using such analyses for clinical translation. Traditional image-based techniques have not yielded the necessary throughput for clinical relevance and translation due to the time required for processing and the limited availability of imaging data from smaller-sized aneurysms, minimizing the utility of such analyses. Therefore, it is imperative that predictions of adverse rupture events incorporate a temporal component (e.g. time-to-event) of medical images that are sub-threshold to aid clinicians in identifying patients that are at high-risk of aneurysm growth toward clinical intervention or rupture. The APC model potentially provides a data-driven clinical decision support tool that compiles clinical data and image-based data from biomechanical and morphological analyses. The reported feature importance compared to Lindquist et al. reveals similar variables that include peak wall stress, and maximum ILT thickness. The APC model was trained with a different set of variables (e.g., our group did not compute ILT stress while Lindquist et al. did not compute various morphological indices that we included in the model), therefore, it is likely that inclusion of exclusion of some variables would result in a different feature importance map. Future studies should consider incorporating such analyses in the clinical management of AAA to potentially reduce surveillance interval or offer early repair when a patient’s predicted prognosis denotes repair or rupture.

Other efforts for predicting growth and rupture of aneurysms have been performed using classification models to identify patient outcomes with mixed results in discriminating patient outcomes^37,38^. Jiang et al.^39^ developed a ML-based growth and remodeling surrogate model to identify local and global changes to aneurysm shape that incorporate temporal changes to a patients aneurysm with clinical data for personalization. The current study used a comprehensive list of clinical, biomechanical, and morphological variables that were later truncated for a more parsimonious model to reduce the potential for overfitting models and removing redundancies within variable types. It is important to note that the computational approaches used here differ from other groups in assembling the FEM. Others reported ML models did not incorporate anisotropic material properties for the aneurysm wall or include the effects of intraluminal thrombus for biomechanical analysis, both potential sources of error in wall stress prediction. However, it is unclear whether the complexity of the model (anisotropic wall material properties and isotropic ILT material properties) is required without interrogating other models. It is unknown whether a minimum threshold for physiologic realism exists without performing computational analyses and may be of interest to the field with further interrogation. Vande Geest et al.^7^ demonstrated that aneurysm wall anisotropy increased wall stresses compared isotropic material properties. In the current study our wall stresses were higher than the wall stresses presented in a recent study performed by Lindquist et al. that used isotropic wall properties (e.g. 18.7 N/cm^2^ vs. 17.2 N/cm^2^ for stable and 23.1 N/cm^2^ vs. 18.8 N/cm^2^, current study vs. Lindquist et al.^38^, respectively). Due to dataset limitations regarding size and diversity of patient outcomes, most studies incorporating ML-based training of models test and train outcomes on using same dataset^36,37^, which is another major limitation. We attempted to alleviate this common pitfall by splitting that dataset into training and testing data (at an 80%/20% ratio) to simulate new data inputs into a trained model to assess performance. The results using this “hold-out validation” was a trained model that was able to classify all three patient outcomes successfully (AUCs > 0.80).

There are limitations to the current study that need to be addressed regarding the development of the APC model. Due to the volume of images delivered from the R3 query, CT image quality based on slice thickness was prioritized for the image-based analyses. The first diagnostic AAA image set within a patient’s history would have been ideal to maximize the time to clinical outcome but was not prioritized as image quality was prioritized. However, the initial diagnosis of AAA is at an unknown point in the natural history of the aneurysm, and with variability of imaging protocols at various hospital sites, it is impossible to standardize the initial image set for all AAAs studied. In addition, three classes of patient outcomes were chosen based on the available follow up for the cohort studied. It is plausible that the ‘stable’ aneurysm cases may eventually grow to a size where clinical intervention, rupture, or patient death unrelated to AAA may have occurred, however, this information was not available within the database that was provided by R3. A potential limitation of the biomechanical analysis was the lack of patient-specific blood pressure as it was unavailable for the study team. However, our group has historically only considered utilizing idealized systolic pressure to standardize by reducing potential noise from unreliable blood pressure measurements during hospital visits. Even though hold-out validation was utilized during the training and testing phase of the model generation, the APC model still relies on internal cross validation. In addition, a class imbalance within the dataset as most of the patients were stable and had no incidence of clinical intervention of rupture. The study team attempted to alleviate potential overfitting effects through splitting and folding the data during training and holding out roughly 25% of the data for validation. Future work to generate a dataset to perform rigorous external cross validation will be explored by interrogating the APC model presented in this study to truly assess the generalizability of the approach. Further, training models to incorporate time-to-event attached to their known outcome can potentially improve clinical utility of this tool.

This study provides, for the first time, a machine learning classification-based methodology utilizing clinical, morphological, and biomechanical data to striate AAA patients based on clinical outcomes. The novel approach enhances the ability for clinicians to understand the patient’s health status through other clinical indices and the physical parameters derived from image-based studies to quantify the biomechanical and morphology. We believe that this classification software tool can be refined to the point that it can better guide clinicians in their management of AAA than just the maximum diameter criterion.

## Conclusion

The APC model demonstrated the ability to striate AAA patients according to outcomes and represents a potentially important step towards the creation of a reliable, noninvasive, objective clinical decision support tool for aneurysm management. Throughout training of the hierarchical levels targeting every combination of categories, it was found that clinical indices alone are insufficient to striate patient outcomes and that imaging-based biomechanical and morphological quantification contributes significantly to ML approaches.

### Supplementary Information

Supplementary Information.

## Data Availability

The retrospective dataset generated and/or analyzed during the current study are not publicly available due to limitations of the scope of the IRB but are available from the corresponding author on reasonable request.

## Abbreviations

AAA: Abdominal aortic aneurysm
AI: Artificial intelligence
CNN: Convolutional neural network
DICOM: Digital imaging and communication
FEA: Finite element analysis
ILT: Intraluminal thrombus
ML: Machine learning
