# Supplementary material for: An artificial intelligence based abdominal aortic aneurysm prognosis classifier to predict patient outcomes
Source: Sci Rep. 2024 Feb 9;14:3390. doi: 10.1038/s41598-024-53459-5 (PMC10858046; doi:10.1038/s41598-024-53459-5)
Supplement: Supplementary file 1 — Supplementary Information. [file 41598_2024_53459_MOESM1_ESM.docx]

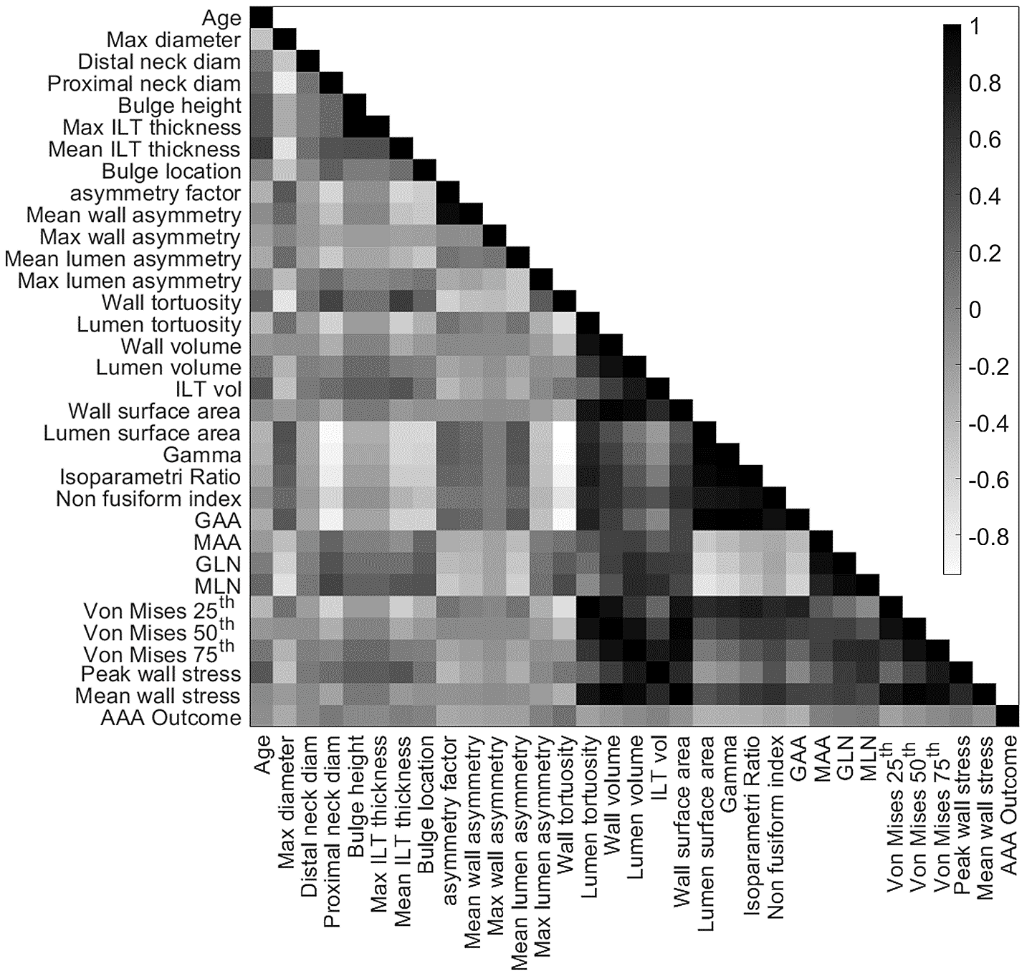


Supplementary Figure 1: Correlation coefficient plot of all morphological and biomechanical indices.


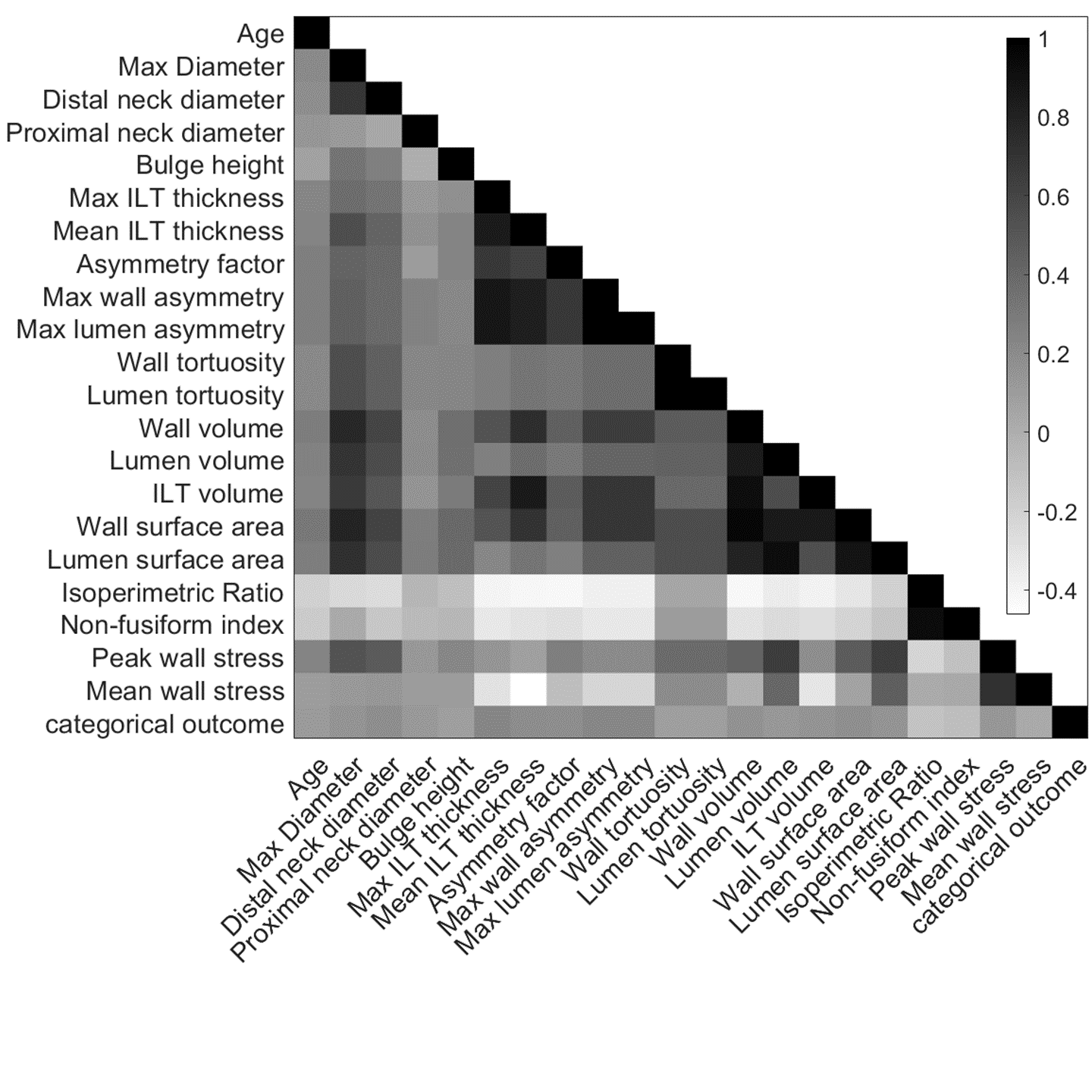


Supplementary Figure 2: Correlation coefficient plot of a subset of morphological and biomechanical indices truncated using 0.02 score of the Gini importance.

**
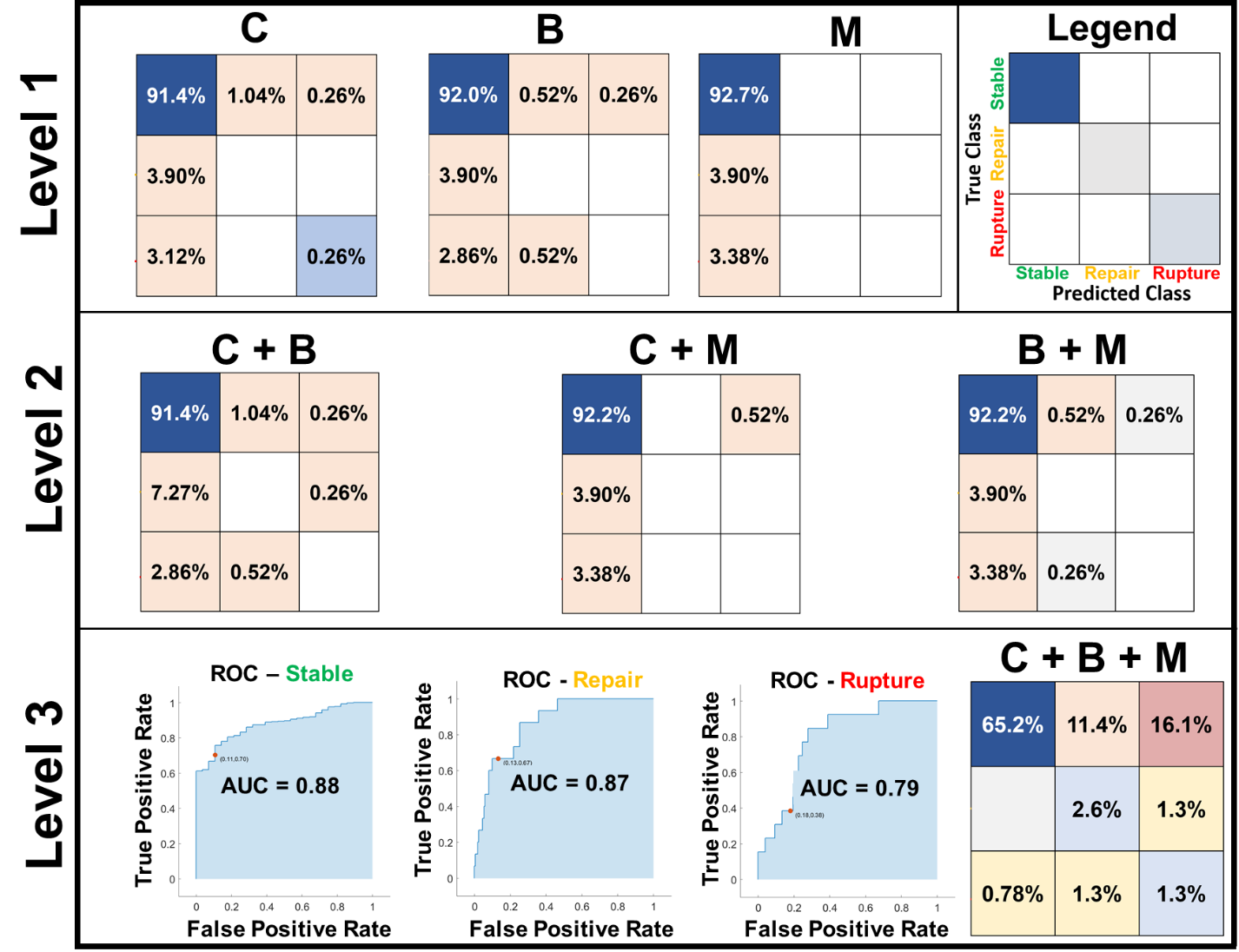
**

Supplementary Figure 3: **Level 1)** Training confusion matrix (CM) results at different levels of individual categories, clinical (C), biomechanical (B), and morphological (M). **Level 2)** CM results for paired categories, and **Level 3)** Receiver operator characteristics plot for stable, repair, and rupture outcomes with respective confusion matrix plot. The confusion matrix reports the predictions of the APC model and their true outcomes.


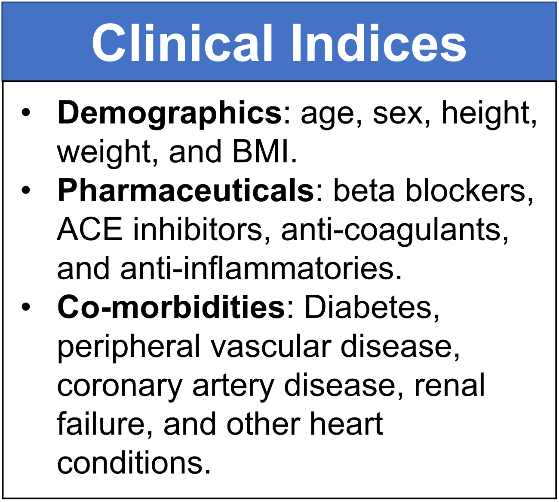


Supplementary Table 1: List of clinical indices that include patient demographics, co-morbidities, and pharmaceutical use.

Supplementary Table 2: Complete list of morphological indices that were calculated from 3D surface reconstructed AAA. The list contains one-, two-, three-, and higher dimensional indices used for machine learning.


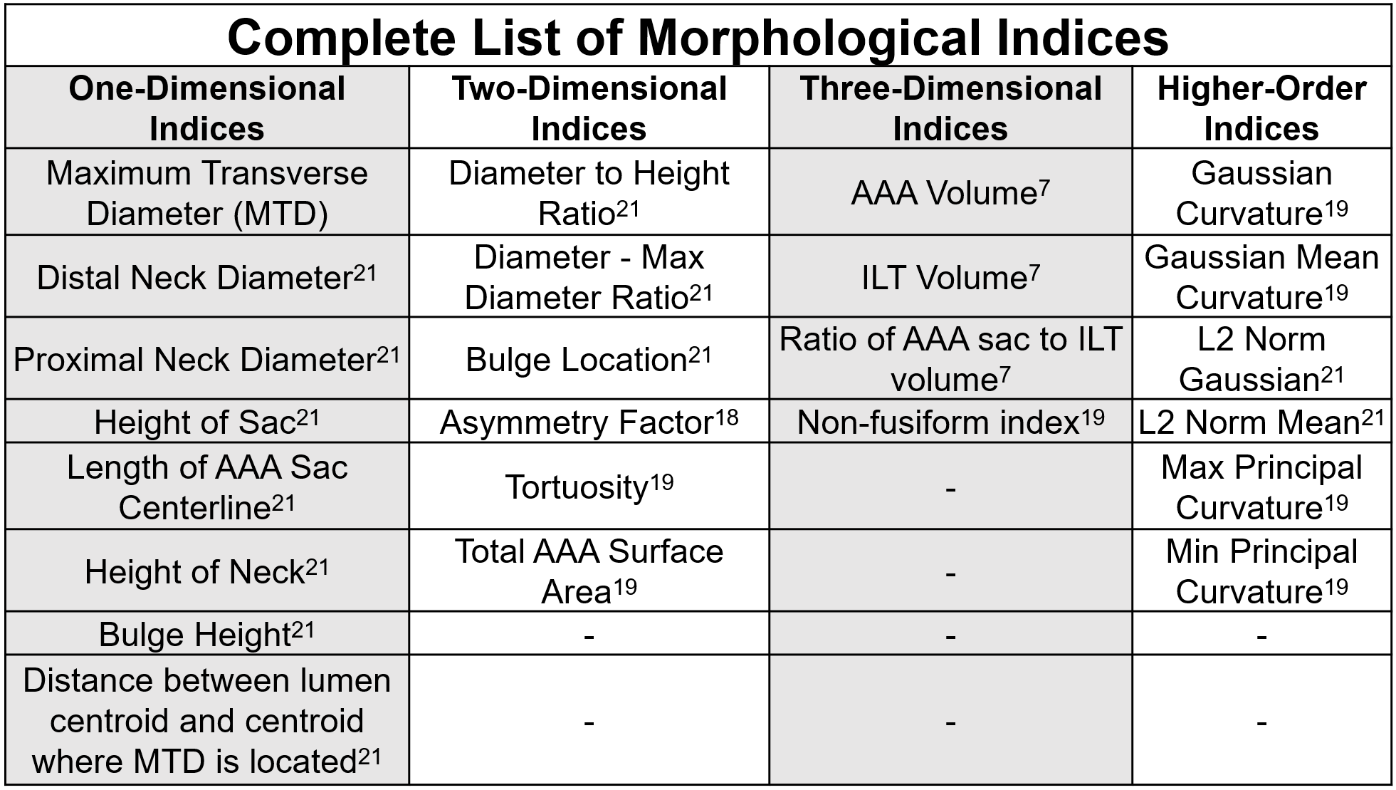
s

| Demographics and clinical indices of patient cohort | | | | | |
| --- | --- | --- | --- | --- | --- |
|  |  |  | Stable (n = 353) | Repair (n = 15) | Rupture (n = 13) |
| Age | | | 67.4 ± 4.60 | 69.9 ± 2.62 | 72.2 ± 2.30 |
| Sex | **Male** | | 71.1% (n = 251) | 80% (n = 12) | 76.9% (n = 10) |
|  | **Female** | | 29.9% (n = 102) | 20% (n = 3) | 23.1% (n = 3) |
| Race | **White** | | 90.9% (n = 321) | 60% (n = 9) | 100% (n = 13) |
|  | **Black** | | 8.53% (n = 30) | 40% (n = 6) | - |
|  | **Asian** | | 0.57% (n = 2) | - | - |
| Chronic Kidney Disease* | | + | 42.5% (n = 150) | 40% (n = 6) | 53.8% (n = 7) |
|  |  | - | 34.6% (n = 122) | 60% (n = 9) | 46.2% (n = 6) |
| Coronary Artery Disease | | + | 70.3% (n = 248) | 46.7% (n = 7) | 84.6% (n = 11) |
|  |  | - | 29.7% (n = 105) | 53.3% (n = 8) | 15.4% (n = 2) |
| Diabetes | | + | 44.2% (n = 156) | 26.7% (n= 4) | 46.2% (n= 6) |
|  |  | - | 55.8% (n = 197) | 73.3 (n = 11) | 53.8% (n = 7) |
| Hyperlipidemia | | + | 81.0% (n = 286) | 93.3% (n = 14) | 92.3% (n = 12) |
|  |  | - | 19.0% (n = 67) | 6.7% (n =1) | 7.7% (n = 1) |
| Hypertension | | + | 94.1% (n = 332) | 100% (n = 15) | 100% (n = 13) |
|  |  | - | 5.9% (n = 21) | - | - |
| Peripheral Artery Disease | | + | 47.0% (n = 166) | 60.0% (n = 9) | 46.2% (n = 10) |
|  |  | - | 53.0% (n = 187) | 40.0% (n = 6) | 53.8% (n = 3) |

Supplementary Table 3: General demographics and clinical indices of AAA patients within each outcome category. Comorbidities are indicated as either a positive history (+) or negative history (-). It should be noted that chronic kidney disease was not assessed for every patient and the percentages given are based on the total sample size of available information.

Supplementary Table 4: Reporting mean and standard deviation range for clinical, biomechanical, and morphological indices. ANOVA F-score and p-value from Tukey’s test.

|  | **Patient Outcome** | | | **ANOVA**  **p-value** | **P-Value** | | |
| --- | --- | --- | --- | --- | --- | --- | --- |
|  | **Stable** | **Repair** | **Rupture** |  | **Stable - Repair** | **Stable - Rupture** | **Repair - Rupture** |
| **Age** | 67.44 ± 4.60 | 69.88 ± 2.62 | 72.22 ± 2.30 | 0.11 | 0.56 | 0.14 | 0.77 |
| **Combined Maximum Diameter (cm)** | 4.39 ± 0.73 | 5.22 ± 0.53 | 5.30 ± 0.48 | 0.01 | 0.07 | 0.06 | 0.99 |
| **Female Maximum Diameter (cm)** | 3.89 ± 1.55 | 4.03 ± 0.46 | 5.31 ± 0.34 | 0.28 | 0.98 | 0.26 | 0.56 |
| **Male Maximum Diameter (cm)** | 4.39 ± 1.45 | 5.22 ± 1.03 | 5.30 ± 0.92 | 0.043 | 0.021 | 0.23 | 0.92 |
| **Distal Neck Diameter (cm)** | 0.73 ± 0.30 | 1.33 ± 0.33 | 1.13 ± 0.33 | 0.00 | 0.00 | 0.05 | 0.67 |
| **Proximal Neck Diameter (cm)** | 1.88 ± 0.21 | 2.00 ± 0.22 | 2.13 ± 0.24 | 0.06 | 0.53 | 0.08 | 0.67 |
| **Sac Height (cm)** | 4.51 ± 1.64 | 6.71 ± 0.64 | 6.09 ± 0.85 | 0.01 | 0.02 | 0.19 | 0.87 |
| **Sac Length (cm)** | 10.46 ± 1.12 | 11.07 ± 1.22 | 12.13 ± 1.13 | 0.02 | 0.55 | 0.02 | 0.43 |
| **Neck Length (cm)** | 21.68 ± 2.37 | 22.80 ± 2.54 | 24.66 ± 2.72 | 0.06 | 0.65 | 0.07 | 0.56 |
| **Neck Height (cm)** | 7.19 ± 1.58 | 5.41 ± 1.02 | 6.86 ± 1.89 | 0.10 | 0.08 | 0.93 | 0.44 |
| **Bulge Height (cm)** | 2.85 ± 1.59 | 3.90 ± 0.70 | 3.95 ± 0.83 | 0.21 | 0.40 | 0.42 | 1.00 |
| **Max ILT thickness (mm)** | 16.26 ± 7.43 | 29.96 ± 2.97 | 30.38 ± 2.97 | 0.00001 | 0.001 | 0.002 | 1.00 |
| **Mean ILT thickness (mm)** | 2.57 ± 1.31 | 4.00 ± 0.67 | 4.92 ± 1.20 | 0.001 | 0.09 | 0.004 | 0.61 |
| **Height Ratio** | 0.62 ± 0.13 | 0.44 ± 0.06 | 0.51 ± 0.07 | 0.01 | 0.01 | 0.23 | 0.74 |
| **Bulge location** | 0.24 ± 0.13 | 0.33 ± 0.07 | 0.30 ± 0.06 | 0.24 | 0.32 | 0.61 | 0.95 |
| **Asymmetry factor** | 0.44 ± 0.33 | 0.98 ± 0.00 | 0.93 ± 0.09 | 0.00 | 0.00 | 0.02 | 0.97 |
| **Wall tortuosity** | 2.86 ± 0.07 | 2.88 ± 0.03 | 2.95 ± 0.05 | 0.06 | 0.84 | 0.05 | 0.37 |
| **Wall Volume (ml)** | 72.74 ± 32.79 | 106.00 ± 29.12 | 120.35 ± 22.76 | 0.01 | 0.13 | 0.03 | 0.83 |
| **Lumen Volume (ml)** | 43.35 ± 15.53 | 61.83 ± 15.39 | 58.15 ± 17.42 | 0.02 | 0.06 | 0.21 | 0.95 |
| **ILT Volume (ml)** | 29.39 ± 21.49 | 44.18 ± 15.13 | 62.20 ± 15.19 | 0.01 | 0.38 | 0.02 | 0.50 |
| **Wall Surface Area cm^2^** | 113.07 ± 27.22 | 144.05 ± 24.30 | 157.21 ± 17.71 | 0.00 | 0.07 | 0.01 | 0.79 |
| **Lumen Surface Area cm^2^** | 89.28 ± 17.09 | 107.53 ± 14.40 | 111.69 ± 18.16 | 0.01 | 0.10 | 0.05 | 0.94 |
| **Gamma** | 2.08 ± 1.33 | 2.86 ± 0.71 | 2.21 ± 0.48 | 0.51 | 0.48 | 0.98 | 0.77 |
| **Isoperimetric Ratio** | 7.52 ± 0.92 | 6.65 ± 0.34 | 6.61 ± 0.35 | 0.04 | 0.16 | 0.17 | 1.00 |
| **Non-fusiform index** | 1.43 ± 0.17 | 1.31 ± 0.06 | 1.29 ± 0.07 | 0.16 | 0.38 | 0.32 | 0.99 |
| **Peak wall stress** | 18.68 ± 3.37 | 23.06 ± 2.40 | 21.95 ± 4.19 | 0.01 | 0.04 | 0.20 | 0.90 |
| **Mean wall stress** | 9.73 ± 1.46 | 10.56 ± 1.10 | 9.70 ± 2.02 | 0.57 | 0.54 | 1.00 | 0.72 |


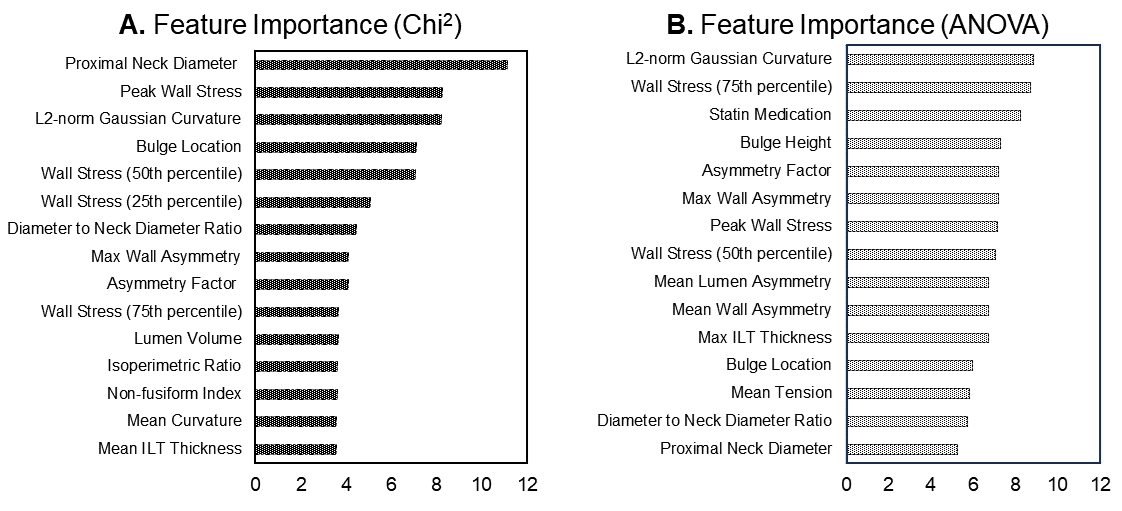


Supplementary Figure 4: Feature importance of the APC model using **A)** Chi-squared test and **B)** variance with ANOVA.
